# Transgenerational soil-mediated differences between plants experienced or naïve to a grass invasion

**DOI:** 10.1002/ece3.716

**Published:** 2013-09-05

**Authors:** Anna Deck, Adrianna Muir, Sharon Strauss

**Affiliations:** Department of Evolution and Ecology, University of CaliforniaDavis, California

**Keywords:** *Achillea millefolium*, *Holcus lanatus*, naïve plasticity, plant invasion, plant–soil feedback, soil microbial community, transgenerational maternal effects

## Abstract

Invasive species may undergo rapid change as they invade. Native species persisting in invaded areas may also experience rapid change over this short timescale relative to native populations in uninvaded areas. We investigated the response of the native *Achillea millefolium* to soil from *Holcus lanatus*-invaded and uninvaded areas, and we sought to determine whether differential responses between *A. millefolium* from invaded (invader experienced) and uninvaded (invader naïve) areas were mediated by soil community changes. Plants grown from seed from experienced and naïve areas responded differently to invaded and uninvaded soil with respect to germination time, biomass, and height. Overall, experienced plants grew faster and taller than their naïve counterparts. Naïve native plants showed negative feedbacks with their home soil and positive feedbacks with invaded soil; experienced plants were less responsive to soil differences. Our results suggest that native plants naïve to invasion may be more sensitive to soil communities than experienced plants, consistent with recent studies. While differences between naïve and experienced plants are transgenerational, our design cannot differentiate between differences that are genetically based, plastic, or both. Regardless, our results highlight the importance of seed source and population history in restoration, emphasizing the restoration potential of experienced seed sources.

## Introduction

Many invasive species both respond to and impose novel selection pressures during the course of an invasion, and provide examples of rapid evolution over short timescales (Sakai et al. 2001; Prentis et al. 2008). Often, native species are locally displaced during an invasion, but, for those that persist, coexistence may expose them to selection arising from invader-caused novel biotic and/or abiotic conditions (Shine 2011). Native species may respond to invaders through plastic responses, changes in allele frequencies, or both (Mooney and Cleland 2006; Phillips and Shine 2006; Strauss et al. 2006). Properties of natives pre- and postinvasion may be substantially different and may represent different resources for use by restoration and conservation biologists in mitigating for impacts of invaders.

In plant communities, invaders may alter soil nutrients or biota in ways that decrease native plant fitness. Callaway et al. (2005) found a correlation between native plants coexisting with the invasive thistle *Centaurea maculosa* and higher tolerance to the allelochemicals produced by *C. maculosa*, suggesting that selection had occurred during the invasion process. Rowe and Leger (2011) also found that native plants from invaded areas were better able to coexist with an invasive grass, becoming more tolerant of competition and showing shifts in several traits such as size and root growth. One indirect change that an invasive plant species may cause over a short timescale is the alteration of soil properties. Indeed, native plants have been shown to have different responses to their coevolved soil communities than to such altered soil communities (Niu et al. 2007; Batten et al. 2008; Mangla et al. 2008). These prior results suggest the potential for selection on native plants by a plant invasion via indirect effects through the soil biotic community.

Differences between plants naïve or experienced with invaders may reflect past selection from invasion. In the study of impacts of invaders on natives, few studies identify whether natives originated from invaded or uninvaded areas (Mealor et al. 2004; Callaway et al. 2005; Lau 2006; Mealor and Hild 2006; Leger 2008; Rowe and Leger 2011) and even fewer studies to date have isolated the response of native plants from invaded or uninvaded origins to soil communities from these same areas (but see Lankau 2012). Environments of parental plants, including competitive environments, may alter the expression of traits in offspring, a process called transgenerational plasticity. Another source of differences between traits of parent and offspring is the result of selection and changes in genotypic composition across generations.

Here, we explore transgenerational effects of maternal plant exposure to an invader on offspring response to soil abiotic properties and biotic communities. We specifically ask: Does history of maternal exposure to invaders affect responses and traits of offspring to soil properties, biotic and abiotic? And, do these responses differ between soils collected from invaded and uninvaded areas?

## The System

In California, *Achillea millefolium* L. (Asteraceae) is one of the few native species that is able to coexist with the widespread invasive perennial grass *Holcus lanatus* (Poaceae), and also one of the few native species that is able to apply a competitive effect on *H. lanatus* (Muir 2009). *Achillea millefolium* is palearctic, and a phylogeographic analysis places BMR populations in a clade that colonized North America via the Bering Land Bridge during the Pleistocene (Ramsey et al. 2008). Over the last century, *H. lanatus*, a Eurasian native, has successfully established on several continents and is now found throughout the United States (Watt 1978; USDA NRCS 2010). Our research is focused on the *H. lanatus* invasion in the California coastal prairie at the University of California, Davis Bodega Marine Reserve (BMR, Sonoma County, CA, 38^o^18′N, 123^o^03′W); where it threatens the native plant community (Elliott and Wehausen 1974; Peart 1989; Kotanen 2004; Thomsen et al. 2006). Where *H. lanatus* invades, it dramatically reduces native species richness (at BMR, uninvaded species richness = 9.4 species ±0.51 SE, and invaded species richness = 6.7 species ±0.27 SE, *P* < 0.0001), and also increases canopy height to more than twice that of uninvaded areas (at BMR, invaded canopy height = 0.81 m ± 0.03 SE, and uninvaded canopy height = 0.30 m ± 0.02 SE, *P* < 0.0001).

Once established, *H. lanatus* can cause substantial biotic and abiotic changes to the soil. Among other alterations to soil biota, soils from *H. lanatus-*dominated areas have lower AMF fungal biomass than soils from nearby uninvaded areas (Innes et al. 2004; Muir 2009) and have higher N content in some portions of the growing season (Muir 2009, Bastow and A. Muir unpubl. data, and see Data S1).

Changes caused by *H. lanatus* to the abiotic and biotic soil environment may influence the interactions between *H. lanatus* and native species, specifically, *A. millefolium*. Given the strong ecological effects of the *H. lanatus* invasion on native plants, it is reasonable to suspect that coexistence of *A. millefolium* with *H. lanatus* requires plastic or genetically based adaptive changes in response to this invasion.

## Methods

### Field collection

In 2006, we collected soil and *A. millefolium* seed in areas of BMR that were either uninvaded or invaded by *H. lanatus*. Invaded sites included areas where *H. lanatus* had been present for 8–60 years (P. Connors, S. Strauss, pers. obs.). Uninvaded sites were located in areas where *H. lanatus* had not been found, but had the capacity to establish; in fact, despite vigorous control efforts at BMR, the vast majority of our uninvaded areas have since been invaded by *H. lanatus* (S. Strauss, P. Connors, pers. obs.).

Seed was mass collected from more than 50 *A. millefolium* individuals in each of the invaded and uninvaded areas and pooled separately according to maternal experience (i.e., parent plants either naïve or experienced to *H. lanatus*). To determine whether initial seed mass differed between invaded and uninvaded areas, we weighed seeds from the mass seed collections of these areas. Seeds are tiny, so seeds within area type were homogenized and then sampled haphazardly in 25 batches of 25 seeds per batch per area. Seeds from uninvaded areas (naïve maternal experience) were significantly heavier than those from invaded areas (experienced maternal experience) [mean mass of individual seed per batch = 0.155 mg experienced (±0.002 SE) and 0.181 mg naïve (±0.002 SE); *P* < 0.001, *t* = 6.51, df = 48].

Within both the invaded and uninvaded sites, soil was sampled from five locations separated by at least five meters. Soil in invaded areas was collected under *H. lanatus* from the top 20 cm of soil in which *H. lanatus* roots are most concentrated (Thomsen and D'Antonio 2007). Soil in uninvaded areas was collected where *A. millefolium* was present, and was collected within the same 20 cm depth profile. Soil was homogenized within invasion type (i.e., *H. lanatus*-invaded sites or uninvaded sites). While we recognize that this homogenization may result in pseudoreplication of soil origin with respect to invasion, one can legitimately treat native and invaded soils as two different soils with which to compare the response of naïve and experienced *A. millefolium* plants, as this was the main focus of our study. For the rest of the methods and results, we refer to soils as invaded or uninvaded, but we recognize (and discuss later) that our ability to attribute differences in response to soils as a result of invaded status is limited.

### Greenhouse methods

The experiment was planted into 600 mL conical Deepots with specific soil treatments. The majority of each pot was filled with a “background soil,” either sterilized invaded or uninvaded soil mixed 1:1 with sterilized sand, and repeatedly autoclaved. Field background soils maintain the texture and the relative amounts of the basic nutrients that might differ across areas. The middle 100 mL of the pots was filled with an isolated soil inoculum that was either invaded or uninvaded and either sterilized or live. In another study, such soil sterilization techniques using BMR soil resulted in at least a 90% decrease in AMF colonization of roots (Bennett et al. 2011).

Into each combination of background soil (sterilized, from either the invaded or uninvaded area) and inocula (inoculum sterilization: either live or sterilized; inoculum origin: from either the invaded or uninvaded area), we planted three *A. millefolium* seeds (seed type: refers to maternal experience as either experienced or naïve to *H. lanatus)*. Each of these 16 treatment combinations was replicated in 12 pots, for a total of 192 pots. If more than one seed germinated, seedlings were culled so that only one remained per pot. To maintain replication, poor germination was supplemented with additional seed or with seedling transplants that had been germinated in the respective soil treatment, and replacement was noted.

Plants were grown for 3 months in a greenhouse and watered to maintain soil moisture. We minimized nutrient differences between treatments by regularly applying non-P fertilizer (0.95 mg/g soil, 20:0:20 NPK fertilizer, equal parts of Ca(NO_3_) and KNO_3_). Plants were harvested after 3 months for measurements of aboveground height and total biomass (above- and belowground dry biomass).

## Analysis

To test for the effects of soil treatment and seed type, we examined time to germination by analysis of variance (ANOVA), total biomass by analysis of covariance (ANCOVA), and height by one-way Welch's ANOVA. All dependent variables were analyzed as a function of the fixed effects (*A. millefolium* seed type [naïve or experienced], inoculum origin [invaded or uninvaded], inoculum sterilization [sterilized or live], and background soil source [invaded or uninvaded]) and their interactions. Four-way interactions were never significant (*P* > 0.9 in all cases) and were dropped from the model. For total biomass, we tested the model with and without seed germination date as a covariate to understand whether any differences among treatments were primarily a function of germination behavior. These two models showed qualitatively identical results, and we present results with germination date in the model to explore effects above and beyond those of germination timing on total biomass. Belowground biomass showed the same patterns as total biomass; for brevity, these analyses are not presented. For height, the germination date covariate was incorporated by using residuals of regression of height on germination date. We then used these germination date-adjusted height values in Welch's one-way ANOVAs. All comparisons used Tukey–Kramer post hoc tests with least square means. Data were analyzed using SAS ver. 9.2, SAS Institute, Cary, NC.

## Results

Experienced and naïve *A. millefolium* plants responded differently to background soil source, inoculum origin, and inoculum sterilization in complex ways. We found both main effects and interactive effects of these soil properties and *A. millefolium* seed type.

### Effects on germination by inoculum origin and sterilization and background soil

**Figure 1:**
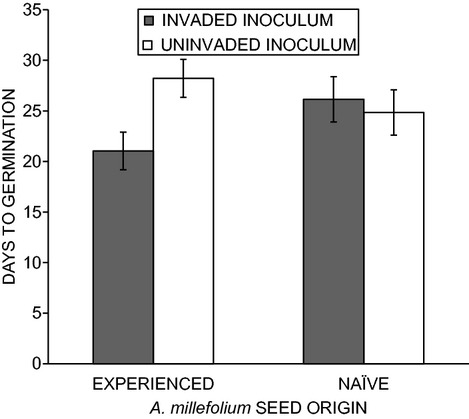
Days to germination of experienced and naïve *Achillea millefolium* seed when planted in inoculum collected from either *Holcus lanatus*- invaded (gray) or uninvaded (white) areas of BMR. Bars are least squares means ± SE.

There were no overall differences in germination timing between seed types; however, there were several interactions with our treatments. Experienced plants germinated ∼7 days earlier when grown with invaded than uninvaded inoculum (Table 1; *P* = 0.04) (Fig. 1). Naïve plants germinated at the same time regardless of inoculum origin. Overall, across both *A. millefolium* seed types, germination occurred 7 days earlier in invaded background soil than uninvaded background soil (Table 1; *P* = 0.001).

**Table 1:** Results of ANOVA of germination date of seed from native plant *Achillea millefolium*

Source	df	*F* value	*P*-value
Seed type	1, 116	0.18	0.67
Inoculum sterilization	1, 116	3.29	0.07
Inoculum origin	1, 116	2.05	0.16
Background soil source	1, 116	11.23	**0.001**
Seed type × inoculum sterilization	1, 116	0.34	0.56
Seed type × inoculum origin	1, 116	4.31	**0.04**
Seed type × background soil	1, 116	0.88	0.35
Inoculum sterilization × inoculum origin	1, 116	1.57	0.21
Inoculum sterilization × background soil	1, 116	0.03	0.86
Inoculum origin × background soil	1, 116	1.21	0.27
Seed type × inoculum sterilization × inoculum origin	1, 116	0.51	0.48
Seed type × inoculum sterilization × background soil	1, 116	0.62	0.43
Seed type × inoculum origin × background soil	1, 116	0.53	0.47
Inoculum sterilization × inoculum origin × background soil	1, 116	13.55	**0.0004**

Seed was collected from either uninvaded areas or areas invaded by the grass *Holcus lanatus* (seed type). Seed was planted into sterilized background soil from invaded or uninvaded sources and a smaller subset of soil inocula that was either sterilized or not (inoculum sterilization) and from uninvaded or uninvaded areas (inoculum origin).

Bold *P*-values are significant at *P* < 0.05.

There was also a significant three-way interaction between background soil source, inoculum origin, and inoculum sterilization on germination (Table 1; *P* = 0.0004) as follows: with invaded background soil, neither inoculum origin nor sterilization affected time to germination for either seed type. For uninvaded background soil, sterilized inoculum regardless of source, also had no effect on germination rate (*P* = 0.89); in contrast, with live inoculum, plants germinated faster with invaded than uninvaded inoculum (*P* = 0.002). These results suggest that live soil biota determines germination behavior in uninvaded, but not invaded, background soils.

### Main effects on biomass

**Figure 2:**
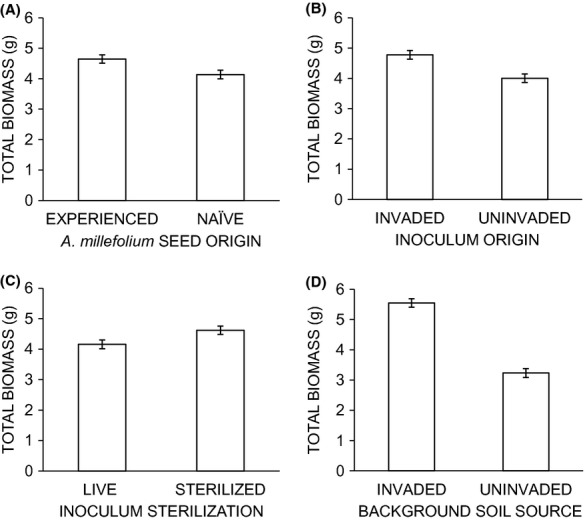
Total *Achillea millefolium* biomass in response to overall effect of background soil source (*Holcus lanatus* invaded or uninvaded) (A), *A. millefolium* seed type (naïve or experienced to *H. lanatus*-invaded soil) (B), inoculum origin (*H. lanatus* invaded or uninvaded) (C), and inoculum sterilization (live or sterilized) (D) (least squares means ± SE). Each factor is significant at *P* < 0.05.

In contrast to germination, there were overall main effects of all treatments – background soil, seed type, inoculum origin, and inoculum sterilization – on total biomass. *Achillea millefolium* grown in invaded background soil had 72% greater total biomass than those grown in uninvaded background soil (Table 2A; *P* < 0.0001) (Fig. 2A), consistent with the observation that invaded areas are more nutrient rich. There were no statistically significant interactions between background soil source and any of the other factors in the model (Table 2A).

**Table 2:** Results of ANCOVA of total biomass (A) and Tukey–Kramer comparisons of experienced and naïve *Achillea millefolium* in each inoculum treatment combination (B)

(A)			
Source	df	*F* value	*P*-value
Germination date (covariate)	1, 171	18.41	**<0.0001**
Seed type	1, 171	6.67	**0.01**
Inoculum sterilization	1, 171	5.40	**0.02**
Inoculum origin	1, 171	14.91	**0.0002**
Background soil source	1, 171	119.16	**<0.0001**
Seed type × inoculum sterilization	1, 171	2.38	0.13
Seed type × inoculum origin	1, 171	1.97	0.16
Seed type × background soil	1, 171	1.11	0.29
Inoculum sterilization × inoculum origin	1, 171	5.94	**0.02**
Inoculum sterilization × background soil	1, 171	3.70	0.06
Inoculum origin × background soil	1, 171	2.79	0.10
Seed type × inoculum sterilization × inoculum origin	1, 171	10.23	**0.001**
Seed type × inoculum sterilization × background soil	1, 171	0.00	1.00
Seed type × inoculum origin × background soil	1, 171	1.13	0.29
Inoculum sterilization × inoculum origin × background soil	1, 171	0.32	0.57

(B)	
Inoculum treatment combination	*P*-value
Invaded sterile	**0.0004**
Invaded live	1.00
Uninvaded sterile	0.85
Uninvaded live	1.00

Seed was collected from either uninvaded areas or areas invaded by the grass *Holcus lanatus* (seed type). Seed was planted into sterilized background soil from invaded or uninvaded sources and a smaller subset of soil inocula that was either sterilized or not (inoculum sterilization) and from uninvaded or uninvaded areas (inoculum origin).

Bold *P*-values are significant at *P* < 0.05.

Across all soil treatments, *A. millefolium* grown from seeds of experienced plants achieved, on average, 12% greater biomass than seeds of naïve plants (Table 2A; *P* = 0.01) (Fig. 2B), despite starting as significantly smaller seeds (see Methods above). This result suggests that faster growth rate may be favored in invaded areas. In addition, soil inoculum taken from invaded areas promoted 19% greater biomass than did soil inoculum from uninvaded areas (Table 2A; *P* = 0.0002) (Fig. 2C) (Data S1). Lastly, across all treatments, inoculum sterilization significantly increased plant biomass by 11% (Table 2A; *P* = 0.02) (Fig. 2D).

### Interactions between soil inoculum, inoculum sterilization, and seed type affect biomass

**Figure 3:**
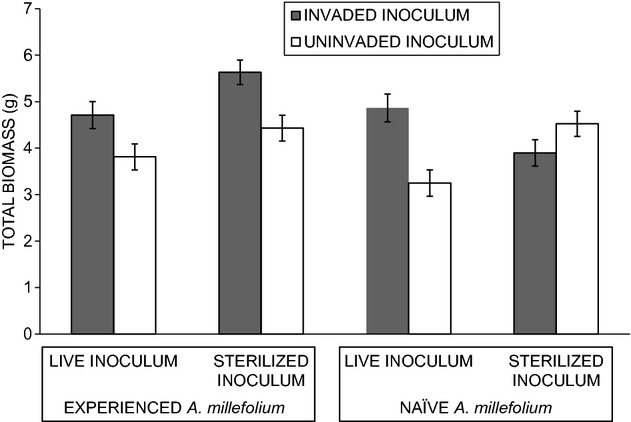
*Achillea millefolium* seed type, inoculum origin, and inoculum sterilization interact to affect total *A. millefolium* biomass. Bars are least squares means ± SE.

A highly significant three-way interaction between inoculum origin, inoculum sterilization, and *A. millefolium* seed type (Table 2A; *P* = 0.002) (Fig. 3) revealed a complex response of naïve and experienced *A. millefolium* to inoculum treatments. Naïve *A. millefolium* achieved 41% more biomass when grown with uninvaded sterilized inoculum than with uninvaded live inoculum (its “home” soil inoculum) (*P* = 0.03), suggesting the possibility of negative soil feedbacks. Interestingly, naïve plants did equally well in sterilized and live inocula from invaded sites (*P* = 0.28), a result, in combination with the above, that suggests that the sterilization process per se was not responsible for better performance in sterilized uninvaded soils. Comparing just across live inocula, naïve *A. millefolium* attained 51% greater biomass in live invaded inoculum than in live uninvaded inoculum (*P* = 0.004).

Experienced *A. millefolium* had equal biomass in live inocula, regardless of inoculum origin (*P* = 0.34); however, when both inocula were sterilized, experienced *A. millefolium* had 27% more biomass with invaded than uninvaded inoculum (*P* = 0.04). This result suggests possible negative soil feedbacks for invader-experienced plants in live invaded soils, as invaders had decreased relative performance in live home soil than sterilized home soil. The magnitude of negative feedbacks (difference in performance between live and sterilized soils) was both greater and significant for naïve (*P* = 0.03) than experienced plants (*P* = 0.28) (41% less in naïve plants and 27% for experienced). Experienced *A. millefolium* achieved greater total biomass than naïve plants when grown in invaded sterilized inoculum (Table 2B; *P* = 0.0004), a result suggesting adaptive transgenerational effects of experience with invaders. In all other inoculum treatments, however, experienced and naïve *A. millefolium* had equal total biomass (Table 2B).

### Effects of seed type and background soil on *A. millefolium* height

Plants grown from seed of experienced *A. millefolium* were significantly taller than those from naïve plants (9% greater) (Table 3; *P* = 0.03). Additionally, *A. millefolium* grew taller in background soil from invaded areas (Table 3; *P* = 0.0001). As with total biomass, there was no significant effect of inoculum origin or inoculum sterilization on *A. millefolium* height (Table 3).

**Table 3:** One-way Welch's ANOVA results of germination date-adjusted height

Source	df	*F* value	*P*-value
Seed type	1, 171	4.93	**0.03**
Inoculum sterilization	1, 171	1.50	0.22
Inoculum origin	1, 171	2.45	0.12
Background soil source	1, 171	15.43	**0.0001**

Seed was collected from either uninvaded areas or areas invaded by the grass *Holcus lanatus* (seed type). Seed was planted into sterilized background soil from invaded or uninvaded sources and a smaller subset of soil inocula that was either sterilized or not (inoculum sterilization) and from uninvaded or uninvaded areas (inoculum origin).

Bold *P*-values are significant at *P* < 0.05.

## Discussion

On the basis of the differential responses of *A. millefolium* plants grown from seed of plants either experienced or naïve to *H. lanatus* invasion, we infer that this invader selects for different phenotypes of *A. millefolium* from those favored in the native, uninvaded community. Moreover, not only were these differences morphological (e.g., selection for greater height and faster growth rates), but experienced and naïve plants also differed in their responses to soils and soil communities, suggesting selection from both biotic and abiotic soil attributes on offspring traits.

Overall, progeny of experienced plants attained greater biomass and height than those of naïve plants. We interpret this result as adaptive transgenerational responses to invasion as the *H. lanatus* canopy is twice as tall in uninvaded areas (see Methods) and because *H. lanatus* has a very rapid growth rate (Bennett et al. 2011). Importantly, experienced and naïve plants responded very differently to soil biota from different sources. When grown in uninvaded soil inoculum, naïve *A. millefolium* grew significantly larger when the inoculum was sterilized than when it was live, suggesting negative feedbacks with soil biota communities in uninvaded areas. Experienced *A. millefolium* were indifferent to live or sterilized inocula from uninvaded areas, and, although they showed a trend toward greater growth when inocula from invaded areas was sterilized, they were not as inhibited as naïve plants by their live home soil biota. We expected naïve *A. millefolium* to show positive soil feedbacks because it is a common, mycorrhizal species (Klironomos 2002) and previous work has shown many native species to prefer uninvaded soil to soil altered by invasion (Batten et al. 2006, 2008; Niu et al. 2007; van der Putten et al. 2007; Mangla et al. 2008). However, we only found evidence for negative soil feedbacks (Bever 1994, 2002; Bonanomi et al. 2005; Kardol et al. 2006; Engelkes et al. 2008).

Although soil sterilization has been shown to increase nutrient levels (e.g., Endlweber and Scheu 2006), several lines of evidence suggest that the disadvantages of live soils to native plants are not nutrient driven. First, we provided a low dose of fertilizer to plants to try to minimize differences in nutrients across live and sterilized soils. Second and most compellingly, sterilization did not increase performance in all cases; experienced *A. millefolium* did not differ in performance when the inoculum was live or sterilized. Likewise, sterilization did not increase performance of naïve *A. millefolium* in invaded inocula. This strong context dependency of the sterilization effect seems more likely to reflect differences in soil biota than changes in nutrients.

Some research has suggested that invasive species have evolved to become less sensitive to soil biota (Seifert et al. 2009; Bennett and Strauss 2013). Our results suggest that native species may, too, be selected to become less sensitive to soil biota when they compete with invaders, a result also found by Lankau (2012) who found that a native plant, *Pilea pumila*, responded to invasion of garlic mustard by reducing dependence on AMF. Experienced *A. millefolium* were less sensitive to soil biota than naïve *A. millefolium*: experienced plants had statistically equal performance in live and sterilized soil inocula from either uninvaded or invaded areas, showing only a trend toward a negative feedback with live inocula from invaded areas. In contrast, naïve *A. millefolium* showed strong negative feedbacks – a 41% reduction in growth – with live soil inoculum collected from uninvaded areas.

Our study cannot distinguish between maternal effects versus genetically based changes in response to soil properties, and these are not mutually exclusive sources of transgenerational effects. Any maternal effects in our experiment would have been transgenerational as our plants were all grown from seed in a common experimental environment. Seed from naïve *A. millefolium* had significantly greater initial mass than experienced *A. millefolium* seed in our study, thus experienced *A. millefolium* grew larger than naïve *A. millefolium* despite provisional differences in initial seed weight. Recently, we have come to appreciate that maternal effects go far beyond provisioning effects. Dyer et al. (2010) showed that transgenerational maternal response to soil conditions increased stress tolerance in seedlings through greater photosynthetic efficiency; a similar plastic response might increase growth rates of experienced *A. millefolium*.

Responses to invasive plants might also have a genetic basis, or the degree of plasticity expressed might have a genetic basis. *Achillea millefolium* is long lived, so if responses to invasion are due to adaptation, the mechanism is likely to be clonal selection in which only well-suited genotypes can survive with *H. lanatus*. Mealor and Hild (2006) showed that, for two native perennial grasses, experienced and naïve populations diverged at a few loci between invaded and uninvaded populations, and circumstantial evidence strongly suggested that natives had evolved in response to selection from an invasive plant. Thus, either or both mechanisms may ameliorate the impact of invasive plants on native plants.

Although we found some evidence for negative soil feedbacks, we did not find any evidence that naïve *A. millefolium* were inherently ill suited for invaded soils; naïve plants did not differ significantly in biomass when invaded inoculum was live or sterilized. Because we did not have true replication of uninvaded and invaded soils (as soils were sampled from five different sites, but then thoroughly mixed to form a standard soil from invaded or uninvaded areas), our ability to attribute differences in response of experienced and naïve plants, or negative soil feedbacks to invasion, per se, is limited. We do, however, demonstrate that experienced and naïve seed sources have significantly different responses to these two different soil sources.

*Holcus lanatus* is a strong interspecific competitor, and many of the phenotypic changes in *A. millefolium* could be due to direct competition: Direct competitive effects of *H. lanatus* on another native plant at BMR, *Erigeron glaucus*, were stronger than soil-mediated effects (Bennett et al. 2011). In our experiment, experienced *A. millefolium* germinated faster in invaded than in uninvaded inoculum and grew taller and larger than naïve plants overall. *Holcus lanatus*, like many introduced grasses in California, has a faster growth rate than native plants (Muir 2009; Bennett et al. 2011). Thus, earlier germination and greater growth rates may be especially important in *A. millefolium* when coexisting with *H. lanatus*. *H. lanatus* canopy is also much taller than that of the native vegetation; this may favor taller native plants in invaded areas, as well as faster growth rates to reach the canopy of the California coastal prairie.

Restoration ecology is beginning to integrate plant–soil feedbacks into ecological restoration programs (Eviner and Hawkes 2008; Heneghan et al. 2008; Kardol and Wardle 2010), as well as appreciates the importance of selecting the correct seed stock for restoration (e.g., Hufford and Mazer 2003). Our research demonstrates the importance of considering seed source and population history used for restoration because of potential adaptation and plasticity seen in *A. millefolium*. Restoration efforts that include replanting with experienced native plant seed may have greater success in previously invaded sites. Experienced A. *millefolium* showed faster germination rates with invaded inocula and greater growth rates based on both height and total biomass. Thus, we expect that plants already experienced to an invader might do better with soil legacies of invasion in restoration.

Previous work has shown differentiation between experienced and naïve populations of a native species in response to a variety of invasion-induced selection pressures (Mealor et al. 2004; Callaway et al. 2005; Lau 2006; Mealor and Hild 2006; Leger 2008; Rowe and Leger 2011; and see Strauss et al. 2006). However, few previous studies investigating native response to invasion-altered soil communities have differentiated between origins of native seed source with respect to their history with the invader (Lankau 2012). This study provides experimental support for rapid change in a native species, as well as the first evidence for transgenerational effects in responses to soil biota. Experiments such as this one are needed to both further our understanding of the potential for native species to respond and adapt to invasions, and to provide valuable insight into the role of transgenerational responses to species coexistence and restoration.

## Supporting Information

**Data S1.** Soil differences between invaded and uninvaded areas.
