# Supplementary material for: Transgenerational soil-mediated differences between plants experienced or naïve to a grass invasion
Source: Ecol Evol. 2013 Sep 5;3(11):3663–71. doi: 10.1002/ece3.716 (PMC3810866; doi:10.1002/ece3.716)
Supplement: Supplementary file 1 [file ece30003-3663-SD1.docx]

**Supplementary Materials**

Supplement 1: Soil Differences Between Invaded and Uninvaded Areas

Soil differences between *Holcus*-invaded and univaded areas are based on soil samples collected from 20 sites under *H. lanatus* canopy or 20 sites from uninvaded areas. A) Discriminant Function Analysis correctly classifies all points and 95% of the variation was explained by the first canonical axis, primarily characterized by invaded sites having greater H, cation exchange capacity (CEC), and lower pH. Soil CEC is positively linked to N and P and other elemental uptake by plants (Bledsoe and Zasoski 1993; Fageria et al 2010). B) Means with asterisks are significantly different between soil types. For units, see table in A.


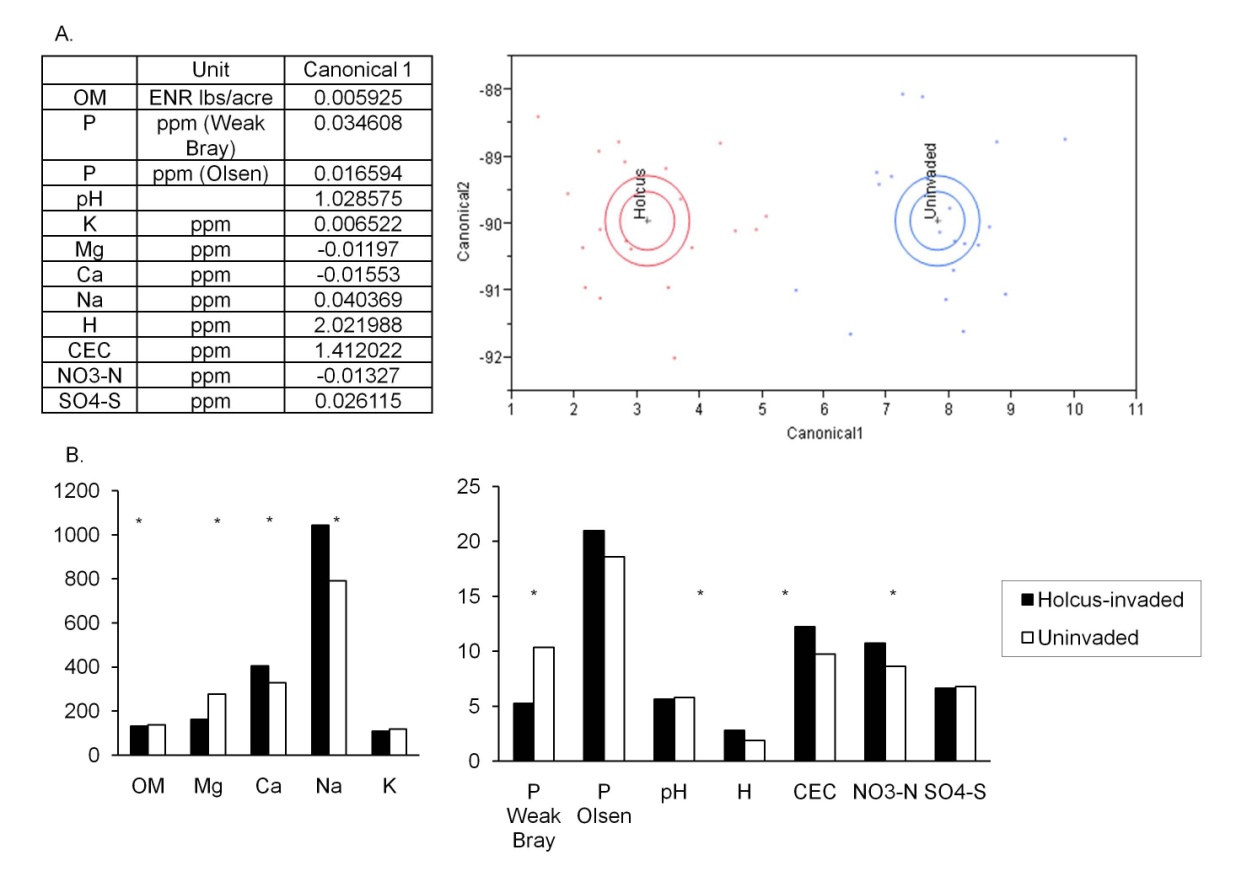


Bledsoe, C.S. and R.J. Zasoski. 1993. Effects of ammonium and nitrate on growth and nitrogen uptake by mycorrhizal douglas-fir seedlings. Plant and Soil 71:445-454.

Fageria, N. K., A. B. Dos Santos, A. Moreira. 2010. Yield, nutrient uptake, and changes in soil chemical properties as influenced by liming and iron application in common bean in a no-tillage system. Communications in Soil Science and Plant Analysis 41:1740-1749.
